# Synthesis, Characterization, and Proton Conductivity of Muconic Acid-Based Polyamides Bearing Sulfonated Moieties

**DOI:** 10.3390/polym15234499

**Published:** 2023-11-23

**Authors:** Carlos Corona-García, Alejandro Onchi, Arlette A. Santiago, Tania E. Soto, Salomón Ramiro Vásquez-García, Daniella Esperanza Pacheco-Catalán, Joel Vargas

**Affiliations:** 1 Instituto de Investigaciones en Materiales, Unidad Morelia, Universidad Nacional Autónoma de México, Antigua Carretera a Pátzcuaro No. 8701, Col. Ex Hacienda de San José de la Huerta, Morelia C.P. 58190, Michoacán, Mexico; carcor93@gmail.com (C.C.-G.); alejandro.onchi@gmail.com (A.O.); 2 Escuela Nacional de Estudios Superiores, Unidad Morelia, Universidad Nacional Autónoma de México, Antigua Carretera a Pátzcuaro No. 8701, Col. Ex Hacienda de San José de la Huerta, Morelia C.P. 58190, Michoacán, Mexico; arlette_santiago@enesmorelia.unam.mx; 3 Centro de Investigaciones Químicas, Instituto de Investigación en Ciencias Básicas y Aplicadas, Universidad Autónoma del Estado de Morelos, Av. Universidad 1001, Cuernavaca C.P. 62209, Morelos, Mexico; tania.soto@uaem.mx; 4 Facultad de Ingeniería Química, Universidad Michoacana de San Nicolás de Hidalgo, General Francisco J. Múgica s/n, Morelia C.P. 58060, Michoacán, Mexico; salomon_vg@yahoo.com; 5 Unidad de Energía Renovable, Centro de Investigación Científica de Yucatán, A.C. Carretera Sierra Papacal-Chuburná Puerto Km 5, Sierra Papacal, Mérida C.P. 97302, Yucatán, Mexico; dpacheco@cicy.mx

**Keywords:** muconic acid, renewable polyamide, sulfonated polymer, ionomer, proton-exchange membrane

## Abstract

Most commercially available polymers are synthesized from compounds derived from petroleum, a finite resource. Because of this, there is a growing interest in the synthesis of new polymeric materials using renewable monomers. Following this concept, this work reports on the use of muconic acid as a renewable source for the development of new polyamides that can be used as proton-exchange membranes. Muconic acid was used as a comonomer in polycondensation reactions with 4,4′-(hexafluoroisopropylidene)bis(*p*-phenyleneoxy)dianiline, 2,5-diaminobencensulfonic acid, and 4,4′-diamino-2,2′-stilbenedisulfonic acid as comonomers in the synthesis of two new series of partially renewable aromatic–aliphatic polyamides, in which the degree of sulfonation was varied. Fourier transform infrared spectroscopy (*FTIR*) and nuclear magnetic resonance (^1^H, ^13^C, and ^19^F-*NMR*) techniques were used to confirm the chemical structures of the new polyamides. It was also observed that the degree of sulfonation was proportional to the molar ratio of the diamines in the feed. Subsequently, membranes were prepared by casting, and a complete characterization was conducted to determine their decomposition temperature (*T_d_*), glass transition temperature (*T_g_*), density (ρ), and other physical properties. In addition, water uptake (*W_u_*), ion-exchange capacity (*IEC*), and proton conductivity (*σ_p_*) were determined for these membranes. Electrochemical impedance spectroscopy (*EIS*) was used to determine the conductivity of the membranes. MUFASA34 exhibited a *σ_p_* value equal to 9.89 mS·cm^−1^, being the highest conductivity of all the membranes synthesized in this study.

## 1. Introduction

The switch to renewable energy and the use of renewable resources is challenging; thus, many industrial processes are designed to work with fossil resources. In addition, in the past, the polymer industry designed materials with the only purpose of meeting the needs and optimizing production costs. This led to several issues, such as environmental pollution and the excessive use of non-renewable resources, among others. Consequently, polymers that can be more easily recycled and that bear some renewable moieties in their structures are currently being investigated [1,2,3].

An alternative technology for clean energy production is the proton-exchange membrane fuel cell (PEMFCs); this device exhibits the qualities of a high power density, easy start-up, scalable size, and low temperature operation; its function is to convert the chemical energy of fuels (such as H_2_, methanol, among others) directly into electrical energy using a proton-exchange membrane (PEM). The desired characteristics of PEMs are usually high proton conductivity; and thermal, chemical, and mechanical stability properties, not allowing the flow of reagents through the membrane and being economically accessible [4,5,6,7].

Accordingly, the PEM is one of the most important parts or PEMFCs. Nafion^®^, marketed by DuPont, is a sulfonated perfluorinated membrane used par excellence in PEMFCs; there are other similar membranes marketed by Dow and Asahi that have also been used as PEMs, however the cost of the membranes prepared from these ionomers is high due to the complicated production process, which limits the wide commercialization of these devices [7,8]. Due to the latter issue, two approaches can be considered to develop materials that can be used as PEMs: the first one is modifying the nafion to improve its properties, but this does not lower the cost of the membrane; the second one is synthesizing polymers with similar chemical structures. It is worth noting that significant progress in fuel cell development has also been made for aromatic polymer membranes [7,9,10,11,12,13] and acid-base blended proton-exchange membranes [14,15,16]. However, at present, almost all commercial synthetic polymers are derived from a small collection of petrochemical feedstocks [17].

In order to circumvent this issue, biomass is considered a very promising alternative to some compounds derived from fossil resources, and several polymers derived from renewable resources are currently being commercialized [18,19,20,21]. When superior mechanical and thermal properties are required, engineering polymers, such as polyamides (PAs), are preferred [22]; in this regard, some polyamides, which are synthesized from renewable raw materials, compete with their petroleum-derived counterparts from an economic perspective [17,22,23].

Muconic acid is a sustainable monomer obtained by biological routes, such as the biofermentation of sugar, lignin, or derivatives [24,25,26,27,28,29]. This compound is a high value-added product because it can be polymerized by different routes. The double bonds present in its structure make it possible to polymerize it by free radicals, while the presence of the two carboxylic acid groups allows polymerization by condensation in combination with diols or diamines [30]. In both types of polymerizations, the polymer backbone presents unsaturations, making it possible for these renewable-based materials to possess improved functionality in terms of their tunable chemical and mechanical properties [24,25,30,31].

Based on the abovementioned ideas, this research explores the synthesis and characterization of new sulfonated polyamides using muconic acid as a renewable comonomer in polycondensation reactions with 4,4′-(hexafluoroisopropylidene)bis(*p*-phenyleneoxy)dianiline (HFDA) and sulfonated diamine 2,5-diaminobencensulfonic acid (DABS) and 4,4′-diamino-2,2′-stilbenedisulfonic acid (DASDA) to produce two series of novel polyamides with a controlled degree of sulfonation, MUFABA and MUFASA, respectively. Their chemical structures are characterized by Fourier transform infrared (*FTIR*) and nuclear magnetic resonance (^1^H, ^13^C, ^19^F-*NMR*) spectroscopies. Electrochemical impedance spectroscopy (*EIS*) is used to determine the proton conductivity of the membrane of new muconic acid-based polyamide membranes. To the best of our knowledge, the use of muconic acid to obtain sulfonated polyamides for proton-exchange membranes has not been investigated. Therefore, this research contributes to the field of green chemistry by providing a sustainable approach to deliver specialty polymer products for alternative energy technologies.

## 2. Materials and Methods

### 2.1. Characterization Techniques

A Bruker Avance III HD (Bruker, Hamburg, Germany) was employed to record ^1^H-*NMR*, ^13^C-*NMR*, and ^19^F-*NMR* spectra at 400, 100, and 376 MHz, respectively. DMSO-*d*_6_ was used as a solvent at a typical concentration of 0.1 g·mL^−1^; hexafluorobenzene (HFB) and tetramethylsilane (TMS) were used as internal standards in this analysis. A Thermo Scientific Nicolet iS10 FTIR spectrometer fitted with an ATR accessory with a diamond crystal was employed to collect 32 spectra and coded for each membrane at 1 × 1 cm and around 400 μm in thickness in a range of 4000 to 650 cm^−1^ at a spectral resolution equal to 4 cm^−1^.

ATGA-DSC equipment (STA449F3 Jupiter; Netzsch, Selb, Germany) was used to determine the thermal stability of the films. For this purpose, the polymeric membrane was placed into an aluminum crucible, which was then placed in a TGA chamber. Subsequently, the sample was treated under a nitrogen flow and heated up from an ambient temperature to 600 °C at 10 °C·min^−1^. The decomposition temperature of the polymers was determined in a TA Instruments Thermogravimetric Analyzer TGA5500 (TA Instruments, New Castle, DE, USA), using samples of around 10 mg, at a temperature range of 30 °C to 800 °C, in a nitrogen atmosphere and a heating rate of 10 °C·min^−1^. A second-generation Bruker D2-Phaser diffractometer was employed to obtain the diffractograms corresponding to polymer films of 2 × 2 cm in size and around 400 μm in thickness. For this purpose, CuK_α_ radiation (1.54 Å) was used at 30 kV, 10 mA, and a 2θ scale from 7 to 70°. A Sartorius analytical balance model, Quintix 124-1 s, was used to determine the density of the membranes through the flotation method using ethanol as the liquid, in ambient conditions. The density measurements were repeated five times and the average of these values was reported for each sample.

Elemental distribution scanning, from the polymers in a membrane form, was performed by Scanning Electron Microscopy using JEOL IT300 equipment (JEOL, Tokyo, Japan). Atomic force microscopy was measured with a Bruker model Multimode 8, using the tapping mode of a Sharp Nitride Lever probe SNL-10 (Bruker, CA, USA). Membranes of 0.5 × 0.5 cm and around 400 μm in thickness were used for SEM and AFM analyses. The Cannon-Ubbelohde viscometer No. 50 was used to determine the inherent viscosity, *η_inh_*, of the polymers. Dissolutions of 0.2 g of each polymer in 100 mL of DMSO were prepared at 30 °C. The *η_inh_* measurements were repeated five times under the given conditions and the average of the values obtained for each polymer sample was reported.

The proton conductivity, *σ_p_*, was determined from *EIS* measurements using a Swagelok cell with 2 current collectors and electrodes of SS 316 using a Biologic VSP potentiostat with FRA, model VMP3B-10, from 1 MHz to 1 Hz at a 50 mV amplitude and open circuit potential under 100% relative humidity at 30 °C; the values were obtained from a Nyquist plot. The mathematical expression used for calculating the proton conductivity is as follows:

(1) $$\sigma_{p}=\frac{l_{m}}{10\times A_{T}\times R_{e}}$$

where *σ_p_* (mS·cm^−1^) represents the proton conductivity, *l_m_* is the membrane thickness (µm), *A_T_* is the cross-section area of the membrane (cm^2^), and *R_e_* is the resistance value of the membrane obtained through the AC impedance method (Ω).

All characterizations were conducted in ambient conditions of humidity and temperature (55% RH and 25 °C, respectively) unless otherwise indicated.

### 2.2. Reagents

Comonomers: muconic acid (MUA), 4,4′-(hexafluoroisopropylidene)bis(*p*-phenyleneoxy)dianiline (HFDA), 2,5-diaminobencensulfonic acid (DABS), and 4,4′-Diamino-2,2′-stilbenedisulfonic acid (DASDA) were used as received. Calcium chloride (CaCl_2_), 1-methyl-2-pyrrolidinone (NMP), triphenylphosphite (TPP), and pyridine (Py) were used in the polycondensation reaction without further purification. Finally, solvents dimethyl sulfoxide (DMSO), dimethylformamide (DMF), and methanol were used as received. All chemical reactants and solvents were acquired from Sigma-Aldrich, Inc.

### 2.3. Synthesis of the Polyamides

**Scheme 1:**
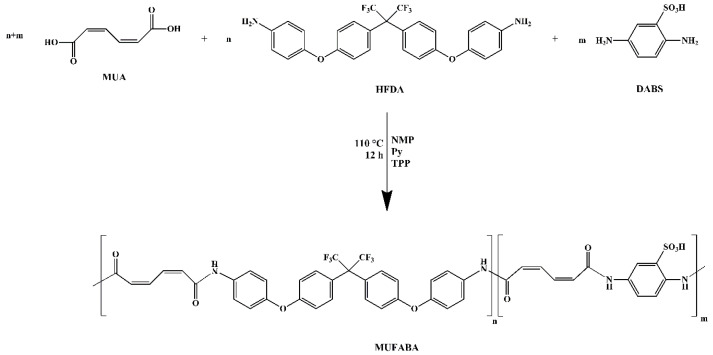
Synthesis route of DABS-containing muconic acid-based polyamides.

**Scheme 2:**
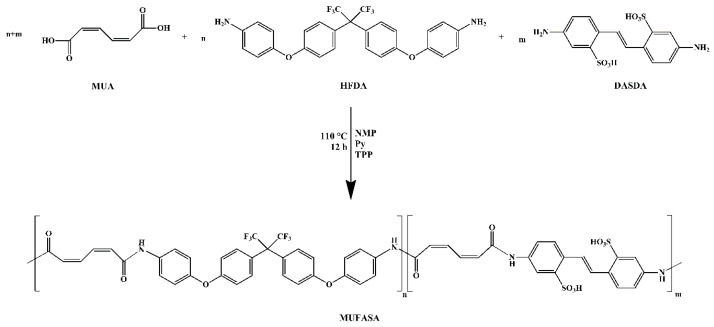
Synthesis route of DASDA-containing muconic acid-based polyamides.

A typical polymerization experiment was conducted as follows: an equimolar mixture of muconic acid and diamines were added to a 50 mL 3-neck flask equipped with a mechanical stirrer in a dry nitrogen atmosphere and according to the amounts shown in Table 1. Then, 2 mL of 1-methyl-2-pyrrolidinone (NMP) and 15 wt% calcium chloride (CaCl_2_) were also added and continuously stirred for 5 min. Then, 0.41 mL of pyridine and 0.41 mL of triphenylphosphite (TPP) were added and maintained with moderate stirring until a fully incorporated mixture was achieved (see Scheme 1 and Scheme 2). Finally, the mixture was heated at 110 °C for 12 h and kept under a constant-stirring condition. Then, the reaction mixture was cooled to room temperature and then precipitated into methanol. The polymer obtained was washed repeatedly with hot water to be purified, and finally it was dried at 100 °C in a vacuum oven for 24 h.

#### 2.3.1. Characterization of Polymer MUFA

^1^H-*NMR* (400 MHz, DMSO-*d*_6_, ppm): δ 10.39 (–NH–, 2H), 7.77–7.75 (aromatic, 4H), 7.34–7.32 (aromatic, 4H), 7.13–7.04 (8H), 6.78 (2H), 6.48–6.23 (2H).

^19^F-*NMR* (376 MHz, DMSO-*d*_6_, ppm): δ −65.9.

#### 2.3.2. Characterization of Polymer MUFABA14

^1^H-*NMR* (400 MHz, DMSO-*d*_6_, ppm): δ 10.37 (–NH–, 8H), 8.36 (aromatic, 1H), 7.99 (aromatic, 2H), 7.77–7.75 (aromatic, 12H), 7.34–7.32 (aromatic, 12H), 7.13–7.04 (aromatic, 24H), 6.76 (8H), 6.48–6.22 (8H).

^19^F-*NMR* (376 MHz, DMSO-*d*_6_, ppm): δ −65.9.

#### 2.3.3. Characterization of Polymer MUFABA24

^1^H-*NMR* (400 MHz, DMSO-*d*_6_, ppm): δ 10.68–10.38 (–NH–, 8H), 8.39 (aromatic, 2H), 7.99 (aromatic, 4H), 7.77–7.75 (aromatic, 8H), 7.35–7.33 (aromatic, 8H), 7.14–7.05 (aromatic, 16H), 6.76 (8H), 6.49–6.23 (8H).

^19^F-*NMR* (376 MHz, DMSO-*d*_6_, ppm): δ −65.9.

#### 2.3.4. Characterization of Polymer MUFABA34

^1^H-*NMR* (400 MHz, DMSO-*d*_6_, ppm): δ 10.68–10.38 (–NH–, 8H), 8.37 (aromatic, 3H), 8.03 (aromatic, 6H), 7.77 (aromatic, 4H), 7.35–7.33 (aromatic, 4H), 7.14–7.05 (8H), 6.76 (8H), 6.48–6.23 (8H).

^19^F-*NMR* (376 MHz, DMSO-*d*_6_, ppm): δ −65.9.

#### 2.3.5. Characterization of Polymer MUFASA14

^1^H-*NMR* (400 MHz, DMSO-*d*_6_, ppm): δ 10.37 (–NH–, 8H), 8.09 (aromatic, 2H), 8.05 (aromatic, 2H), 7.77–7.75 (aromatic, 12H), 7.64 (aromatic, 2H), 7.46 (CH=CH, 2H), 7.34–7.32 (aromatic, 12H), 7.13–7.04 (aromatic, 24H), 6.76 (8H), 6.48–6.23 (8H).

^19^F-*NMR* (376 MHz, DMSO-*d*_6_, ppm): δ −65.9.

#### 2.3.6. Characterization of Polymer MUFASA24

^1^H-*NMR* (400 MHz, DMSO-*d*_6_, ppm): δ 10.49–10.39 (–NH–, 8H), 8.11 (aromatic, 4H), 8.02 (aromatic, 4H), 7.76 (aromatic, 8H), 7.64 (aromatic, 4H), 7.46 (CH=CH, 4H), 7.34–7.32 (aromatic, 8H), 7.13–7.05 (aromatic, 16H), 6.74 (8H), 6.48–6.23 (8H).

^19^F-*NMR* (376 MHz, DMSO-*d*_6_, ppm): δ −65.9.

#### 2.3.7. Characterization of Polymer MUFASA34

^1^H-*NMR* (400 MHz, DMSO-*d*_6_, ppm): δ 10.50–10.40 (–NH–, 8H), 8.11 (aromatic, 6H), 8.01 (aromatic, 6H), 7.77–7.75 (aromatic, 4H), 7.66–7.62 (aromatic, 6H), 7.45 (CH=CH, 6H), 7.34–7.32 (aromatic, 4H), 7.12–7.04 (8H), 6.76 (8H), 6.48–6.23 (8H).

^19^F-*NMR* (376 MHz, DMSO-*d*_6_, ppm): δ −65.9.

### 2.4. Membrane Preparation, Ion-Exchange Capacity, and Water Uptake

Membranes were cast from polymeric DMSO solutions at 60 °C. The solution was filtered and poured onto a glass plate, and the solvent was slowly evaporated in a controlled DMSO atmosphere. Then, the membranes were subjected to treatment as described in the literature [32]. This treatment consisted firstly in the removal of the residual solvent by using methanol and deionized water, followed by activation with 1.0 N of hydrochloric acid. Finally, the membranes were dried under vacuum conditions at 150 °C for 24 h. The average thickness of the films was around 400 μm.

The experimental ion-exchange capacity of the polymer membrane was assessed, as described in the literature [32], by the titration method using the following mathematical expression:

(2) $$IEC=\frac{V\times M}{W_{dry}}$$

where *IEC* (meq·g^−1^) represents the ion-exchange capacity, *V* (mL) represents the volume of the NaOH solution used in the titration, *M* is the molarity of the solution, and *W_dry_* (g) is the mass of the dried membrane.

The theoretical ion-exchange capacity was calculated considering the complete incorporation of monomers and according to the following mathematical expression:

(3) $$IEC_{Theo}=\frac{n_{MS}\times 1000\times sg}{w_{MUA}+w_{HFDA}+w_{MS}}$$

where *IEC_Theo_* (meq·g^−1^) represents the theoretical ion-exchange capacity, *n_MS_* (mol) represents the moles of the sulfonated diamine present on the polymer, *sg* is the number of sulfonic groups corresponding to the DABS or DASDA sulfonated monomers, *w_MUA_* (g) is the weight of monomer MUA, *w_HFDA_* (g) is the weight of fluorinated monomer HFDA, and *w_MS_* (g) is the weight of sulfonated monomers DABS or DASDA.

The water uptake, *W_u_*, of the polymer membrane was estimated, as described in the literature [33], by gravimetric measurements employing the mathematical expression given below:

(4) $$W_{u}=\frac{W_{wet}-W_{dry}}{W_{dry}}\times 100$$

where *W_u_* (%) represents the water uptake, *W_wet_* (g) is the mass of the hydrated membrane, and *W_dry_* (g) is the mass of the dried membrane.

## 3. Results and Discussion

Partially renewable muconic acid-based polyamides with an increasing degree of sulfonation were successfully prepared by a polycondensation reaction employing muconic acid as a renewable monomer and 4,4′-(hexafluoroisopropylidene)bis(*p*-phenyleneoxy)dianiline (HFDA) as a fluorinated comonomer, as well as 2,5-diaminobencensulfonic acid (DABS) and 4,4′-diamino-2,2′-stilbenedisulfonic acid (DASDA) as sulfonated comonomers (see Scheme 1 and Scheme 2, respectively). The roles of DABS and DASDA in this study were to incorporate sulfonic acid groups into the polymer backbone, thus endowing the resulting polymer with proton-exchange properties suitable for being used as a PEM. In addition, the use of both diamines allowed us to elucidate the effect of the number of sulfonic acid groups per repeating unit on the overall property balance.

**Figure 1:**
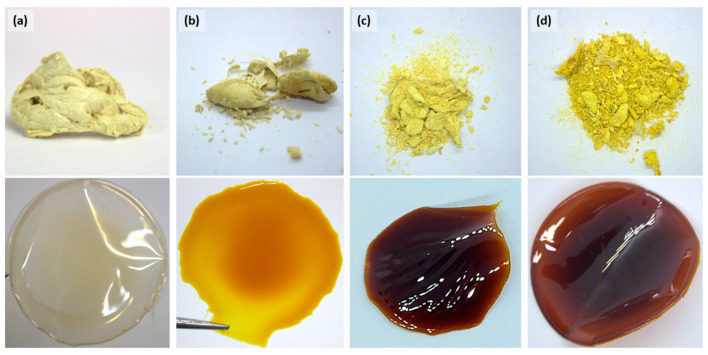
Photographic images of the synthesized raw muconic acid-based polyamides (**top**) and their corresponding polymeric membranes (**bottom**): (**a**) MUFA, (**b**) MUFABA14, (**c**) MUFABA24, and (**d**) MUFABA34.

**Figure 2:**
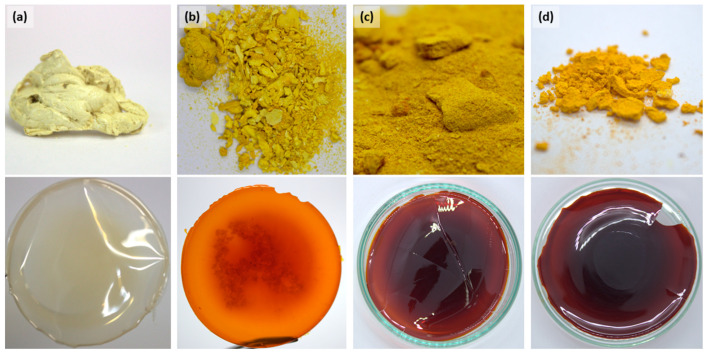
Photographic images of the synthesized raw muconic acid-based polyamides (**top**) and their corresponding polymeric membranes (**bottom**): (**a**) MUFA, (**b**) MUFASA14, (**c**) MUFASA24, and (**d**) MUFASA34.

It could be seen that the degree of sulfonation, *DS*, was effectively adapted by adjusting the molar ratio of diamines fed at the beginning of the reaction. Images of the raw DABS- and DASDA-containing partially renewable polyamides synthesized in this study are presented in Figure 1 and Figure 2, respectively. The non-sulfonated polyamide MUFA yielded yellow fibers and the sulfonated polyamide MUFABA series (Figure 1b–d) afforded fibers with coloration ranging from banana yellow to pineapple yellow, according to the increment in the sulfonation degree. In the same way, the sulfonated MUFASA series (Figure 2b–d) afforded fibers with coloration ranging from banana yellow to golden ochre. From these figures, it also can be seen that the raw material size decreases as the DS increases. According to the latter, the particle size is larger for the DABS-containing polyamides than for the MUFASA polyamides obtained from the same feed ratio. Polymeric membranes were prepared by casting and dissolving the polymers in DMSO. The membrane MUFA, Figure 1d, is transparent in appearance and quite resistant when touched; when the feed amount of sulfonated diamine increases in the polyamides, MUFABA or MUFASA series, the membranes are opaque with an orange-brown color that progressively intensifies as the *DS* increases.

**Figure 3:**
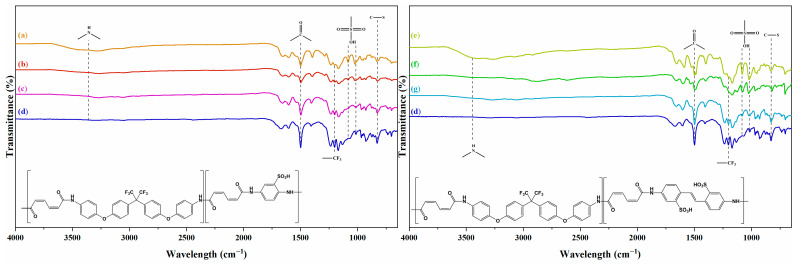
*FTIR* spectra of the muconic acid-based polyamides: (**Left**): (a) MUFABA34, (b) MUFABA24, (c) MUFABA14, (d) MUFA; (**Right**): (e) MUFASA34, (f) MUFASA24, (g) MUFASA14, and (d) MUFA.

The chemical structures of the new muconic acid-based polyamides were first confirmed by *FTIR* spectroscopy. The *FTIR* spectra of these new polyamides are shown in Figure 3; on the left side, the *FTIR* spectra of the series of polyamides containing DABS can be observed, while on the right side, those corresponding to the series of polyamides containing DASDA are shown. In both series, the characteristic absorption band for the N–H bond is observed at around 3300 cm^−1^. The band associated with the amide carbonyl groups (–CONH–) is displayed close to 1660 cm^−1^. The absorption band of C=C is exhibited at around 1500 cm^−1^. The band attributed to the C–F bond can be observed at around 1200 cm^−1^; this signal is more intense in the MUFA polymer, and it decreases as the concentration of the sulfonic acid increases. The presence of the sulfonic acid groups in the polymer backbone is indicated by the absorption bands shown at around 1086 and 1020 cm^−1^, which correspond to the asymmetric and symmetric O=S=O stretching vibrations of the –SO_3_H groups. It should be noted that these signals increase as the concentration of these sulfonic groups increase. The signal observed at around 3400 cm^−1^ can be attributed to the moisture present in the samples, which correlates with a higher concentration of sulfonic groups that makes these polymers more hydrophilic.

**Figure 4:**
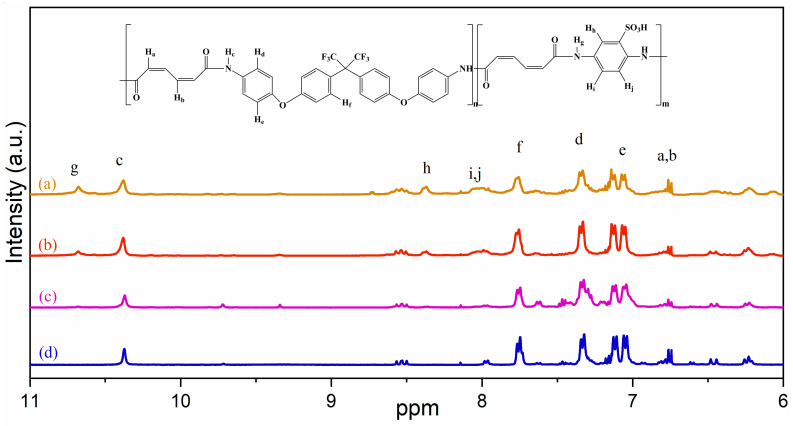
^1^H-*NMR* spectra of the muconic acid-based polyamides: (a) MUFABA14, (b) MUFABA24, (c) MUFABA34, and (d) MUFA. Lower case letters correspond to the protons indicated in the chemical structure.

**Figure 5:**
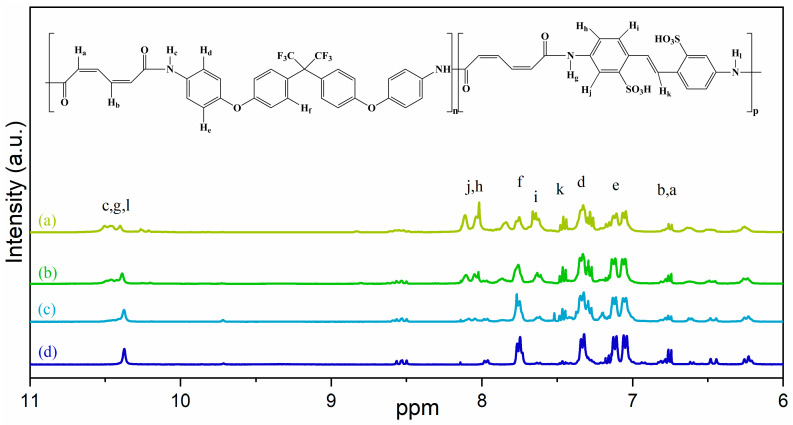
^1^H-*NMR* spectra of the muconic acid-based polyamides: (a) MUFASA34, (b) MUFASA24, (c) MUFASA14, and (d) MUFA. Lower case letters correspond to the protons indicated in the chemical structure.

*NMR* spectroscopy was performed to confirm the chemical structures and compositions of the novel muconic acid-based polyamides, thus corroborating the results obtain by *FTIR*. The ^1^H-*NMR* spectra of the MUFABA and MUFASA polyamide series are shown in Figure 4 and Figure 5, respectively. This analysis indicated that the ratio of the proton integration areas agreed quite well with the expected chemical structures of the polyamides. For instance, in Figure 4, a single signal ascribed to the proton attached to the amide group (H_c_) in the polyamide MUFA can be observed at δ = 10.39 ppm. For the DABS-containing polyamide series, the proton attached to the amide group displays two signals, in δ ≈ 10.68 (H_g_) and 10.38 ppm (H_c_), and it can also be seen that the new signal at 10.68 ppm increases as the concentration of DABS in the polymer increases. Likewise, the signals observed in the region of 8.4–7.9 ppm are attributed to the aromatic protons in the DABS moiety (H_h_, H_i_, H_j_). The signals attributed to the incorporation of the HFDA monomer appear at around δ ≈ 7.7 (H_f_), 7.3 (H_d_), and 7.11–7.04 ppm (H_e_), while the olefinic proton signals ascribed to the incorporation of the muconic acid monomer (H_a_, H_b_) are observed at around 6.8–6.7 ppm; these monomer-derived signals are common for all polyamides synthesized in this study. Finally, in Figure 5, the DASDA-containing polyamide series presents multiple signals attributed to the proton attached to the amide group, in δ ≈ 10.50 (H_g_, H_l_) and 10.40 ppm (H_c_). Moreover, the signals observed in the region of 8.1–7.6 ppm are attributed to the aromatic protons in the DASDA moiety (H_h_, H_i_, H_j_, H_k_).

The intensity of these signals varied progressively according to the fluorinated/sulfonated diamine feed molar ratio. The triplet peaks at around δ ≈ 8.5 ppm in the ^1^H-*NMR* spectra were attributed to pyridine, a reactive used in the synthesis of these polyamides, which was not completely removed after the purification procedure. The ratio between the proton integration areas that remained unchanged in the polyamide MUFA and the series of sulfonated polyamides, MUFABA and MUFASA, were employed to calculate the *DS*. The values of *DS*, determined from the ^1^H-*NMR* spectra, for the muconic acid-based polyamides MUFABA14; MUFABA24; and MUFABA34 were found to be 24.3%; 48.7%; and 72.2%, respectively, while those of the polyamides MUFASA14; MUFASA24; and MUFASA34 were found to be 22.8%; 46.1%; and 70.6%, respectively. These *DS* values were close and could be correlated to the diamine ratio established in the feed values (HFDA, and DABS or DASDA) of the new polyamides, which were 25%, 50%, and 75% with respect to the sulfonated diamine, for each series, respectively (see Table 1). These values indicated that the incorporation of sulfonic groups was effectively controlled.

**Figure 6:**
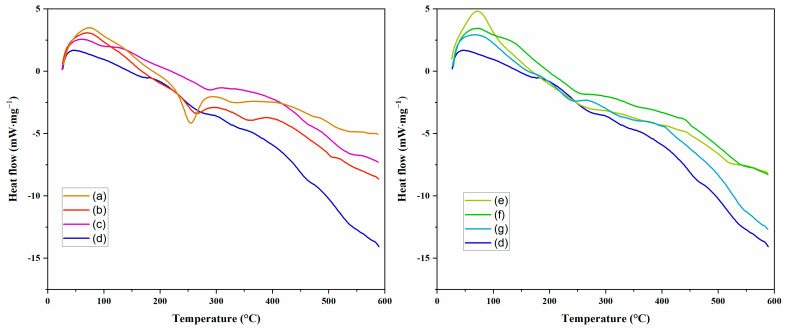
*DSC* thermograms of the DABS-containing polyamide series (**left**): (a) MUFABA34, (b) MUFABA24, (c) MUFABA14, and (d) MUFA, and the DASDA-containing polyamide series (**right**): (e) MUFASA34, (f) MUFASA24, (g) MUFASA14, and (d) MUFA.

The *T_g_* of the polymers was determined by *DSC*, and their thermograms are shown in Figure 6; those corresponding to the DABS-containing polyamide series are displayed on the left side, while those corresponding to the DASDA-containing polyamide series are shown on the right. The DSC curves present some features lower than 200 °C; those observed around 100 °C are attributed to the humidity adsorbed by the membranes, while those observed near 200 °C are attributed to the residual solvent (DMSO) used in the membrane casting. Therefore, the glass transition temperature of the polymer was considered to be the first characteristic above 200 °C. It can be seen that the *T_g_* for the non-sulfonated polymer MUFA is about 241 °C, a high *T_g_* that is ascribed to the rigidity of the main chain caused by the aromatic rings of the HFDA monomer. It was found that for the MUFABA series, the *T_g_* remained almost the same. The *T_g_* values for MUFABA14, MUFABA24, and MUFABA34 were found to be 244 °C, 240 °C, and 241 °C, respectively. The latter can be attributed to two opposite effects. On the one hand, as DS increases, the ratio of fluorinated/sulfonated diamine decreases, and, in turn, so does the rigidity of the polymer chains. On the other hand, the incorporation of sulfonic acid groups creates strong ionic interactions between polymeric chains, increasing their rigidity. This contrasting behavior results in a negligible change in *T_g_* as the quantity of the sulfonated moiety is varied in the polymer. It was also found that for the MUFASA series, the *T_g_* increased as the *DS* increased. The *T_g_* values for MUFASA14, MUFASA24, and MUFASA34 were found to be 217 °C, 229 °C, and 232 °C, respectively. This increase in the *T_g_* value of the MUFASA series could be due to the higher concentration of sulfonic groups in the polymer backbone, which inhibited the relaxation process of polymer main chains as a result of strong ionic interactions between them.

**Figure 7:**
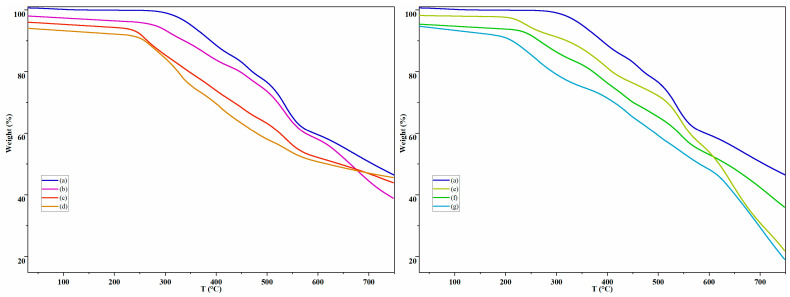
*TGA* thermograms of the DABS-containing polyamide series (**left**): (a) MUFA, (b) MUFABA14, (c) MUFABA24, and (d) MUFABA34, and the DASDA-containing polyamide series (**right**): (a) MUFA, (e) MUFASA14, (f) MUFASA24, and (g) MUFASA34.

The thermal stability of polyamides was studied by *TGA* under a nitrogen atmosphere. The thermograms are presented in Figure 7 with a 2% offset between each thermogram. It can be observed that the polymer MUFA exhibits a decomposition temperature, *T_d_*, of about 322 °C. For the MUFABA series, the *T_d_* values observed were 318 °C, 303 °C, and 273 °C for MUFABA14, MUFABA24, and MUFABA34, respectively, while for the MUFASA series, the *T_d_* values observed were 287 °C, 272 °C, and 276 °C for MUFASA14, MUFASA24, and MUFASA34, respectively. From these results, it is clear that the introduction of labile groups, such as –SO_3_H, into the macromolecular architecture is reflected by a lower *T_d_* value.

**Figure 8:**
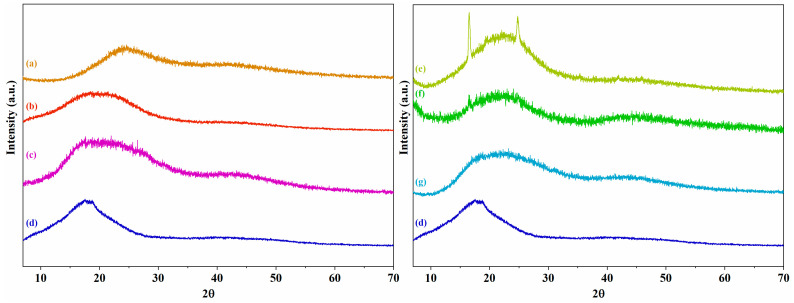
*XRD* patterns of the DABS-containing polyamide series (**left**): (a) MUFABA34, (b) MUFABA24, (c) MUFABA14, and (d) MUFA, and the DASDA-containing polyamide series (**right**): (e) MUFASA34, (f) MUFASA24, (g) MUFASA14, and (d) MUFA.

The mean intersegmental distance or *d-spacing* between the polymer chains was calculated by the Bragg’s equation, $d_{i}=\frac{n\lambda }{2sin\left(\theta \right)}$; the angle was determined at the maximum intensity of the amorphous peak. In general, the *XRD* technique showed patterns characteristic of amorphous polymeric materials, and the absences of narrow peaks showed that these samples did not exhibit any degree of crystallinity, with the exception of the MUFASA34 sample, in which the highest quantity of the DASDA monomer incorporated seemed to induce a certain degree of crystallinity in the resulting polymer (see Figure 8). The maximum reflective intensity was observed at interval of 2θ ≈ 17.5–24.6°. This angle increased as the degree of sulfonation increased.

The density, *ρ*, of the new polyamide membranes was determined by the flotation method using ethanol as the liquid, at room temperature. The values, shown in Table 2, were found to range from 1.38 to 1.45 g·cm^−3^. The inherent viscosity (*η_inh_*), also shown in Table 2, presents values ranging from 0.352 to 0.201 dL·g^−1^. It can be seen that the lowest inherent viscosity is that of the MUFASA34 polymer, indicating that the chain size of this polymer is smaller than that of the other polymers. The *η_inh_* values obtained are in the range of other synthetic polymers [34,35]. The *η_inh_* values of the DABS-containing polyamides are very similar to each other, whereas the *η_inh_* values of the DASDA-containing polyamides decrease as the *DS* increases; it is likely that the higher amount of DASDA in the feed causes the insolubility of the reactants in the polymer solution, affecting the growth of the polymer.

**Figure 9:**
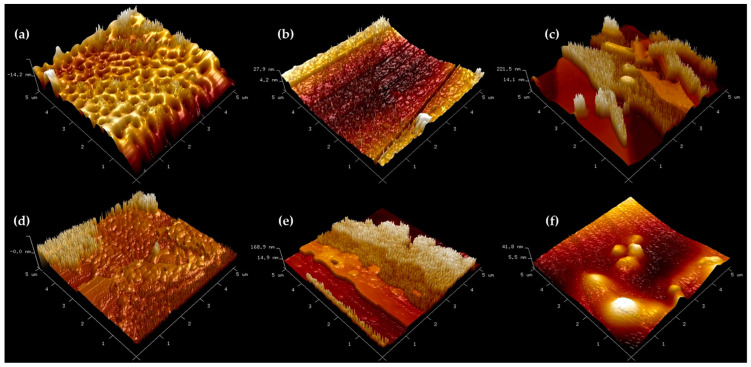
Three-dimensional *AFM* micrographs (5 × 5 μm) of (**a**) MUFABA14, (**b**) MUFABA24, (**c**) MUFABA34, (**d**) MUFASA14, (**e**) MUFASA24, and (**f**) MUFASA34.

The morphology of the membranes was observed by *AFM* and the topographic images obtained by this technique, in tapping mode, are presented in Figure 9. The morphology of the MUFABA14 sample is mainly characterized by voids and a few areas with protuberances that can be associated with hydrophobic fluorine-rich regions. The MUFABA24 sample presents a homogeneous surface morphology, in which the existence of voids or large protuberances is not appreciated; this can be attributed to the balance between the hydrophobic and hydrophilic regions created by the sulfonic acid groups. In contrast, the MUFABA34 sample shows a quite irregular surface morphology consisting of plateaus immersed in regions with protuberances of different sizes that can be attributed to the higher number of –SO_3_H groups in this entire series of polyamides, which causes the disruption of the chain-packing process. The topology of MUFASA14 is mainly characterized by a regular surface formed by small protuberances that lacks voids and resembles that of MUFABA14. The MUFASA24 sample exhibits a surface morphology that is composed of different plateaus and some areas with pinnacles in which phase segregation can be noticed. Finally, the MUFASA34 sample shows a surface on which quite large protuberances can be seen. Due to the high *DS* of this polyamide, it is likely that large hydrophilic regions are formed and these begin to segregate from the hydrophobic regions, giving rise to large clusters. These observations indicate the existence of phase separation, which is a fundamental aspect to achieve high proton conductivity, since the segregation of hydrophilic and hydrophobic phases builds the channels through which ionic transport is conducted.

**Figure 10:**
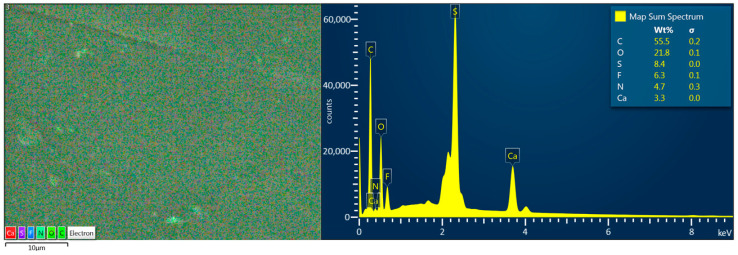
*SEM* image and *EDS* spectra of MUFABA34.

**Figure 11:**
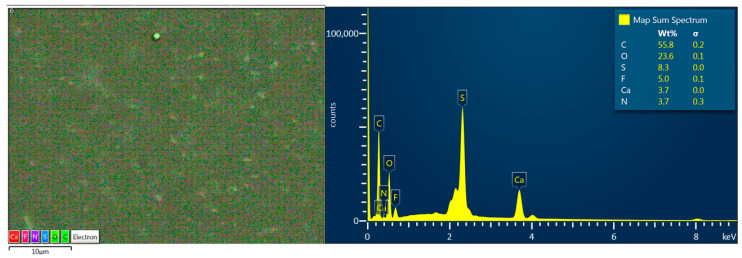
*SEM* image and *EDS* spectra of MUFASA34.

Scanning electron microscopy (*SEM*) and energy-dispersive X-ray spectroscopy (*EDS*) were employed to study the surfaces of the polyamide samples with the highest content of sulfonic acid groups in each series. Representative results are shown in Figure 10 and Figure 11, corresponding to polymers MUFABA34 and MUFASA34, respectively. The *SEM* images show the heterogeneous surface texture and morphology for these partially renewable ionomers that can be attributed to phase segregation. Furthermore, *EDS* was employed to estimate the chemical composition of the polymers. As can be seen, both polyamide samples mainly show the presence of carbon (C), oxygen (O), sulfur (S), fluorine (F), nitrogen (N), and calcium (Ca). These results, specifically the sulfur weight percent and fluorine weight percent determined by *EDS*, are in agreement with those expected from the fluorinated/sulfonated diamine feed molar ratio.

**Figure 12:**
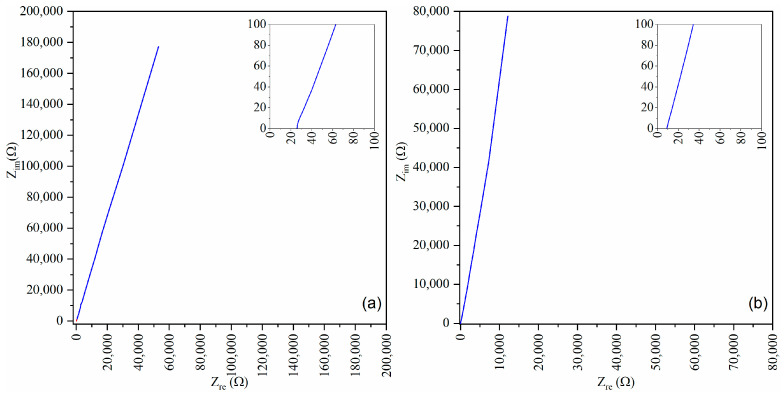
Nyquist plots of (**a**) MUFABA34 and (**b**) MUFASA34. Inset: zoomed-in view of the Nyquist plots in the high-frequency-region membrane.

The electrochemical impedance spectroscopy (*EIS*) measurements of the sulfonated membranes were performed under 100% relative humidity at 30 °C in the range of 1 MHz to 1 Hz; Nyquist plots of the most sulfonated membranes of each series, MUFABA34 and MUFASA34, are shown in Figure 12. The resistance, *R_e_*, of the membranes was calculated using the “ZFit” function of EC-Lab software V11.27 from the intersection with the abscissa axis, which corresponds to the resistance value, as can be observed in the insets of Figure 12; all measures are repeated three times in the given conditions, and the averages of the results obtained are reported. Then, the proton conductivity, *σ_p_*, was calculated by Equation (1), and the results are presented in Table 3. It can be observed that the *σ_p_* value increases as the sulfonic acid group concentration increases. The *σ_p_* values for the MUFABA polyamide series were found to range from 0.027 to 1.608 mS·cm^−1^, while those of the MUFASA polyamide series were found to range from 0.367 to 9.895 mS·cm^−1^. It is important to mention that, when comparing a polyamide from the MUFABA series with one from the MUFASA series with similar *DS* values, the latter actually contains twice as many –SO_3_H groups as the former, because the diamine DASDA, which gives rise to the MUFASA series, contains one more –SO_3_H group than the diamine DABS, which gives rise to the MUFABA series. For this reason, the MUFASA34 membrane, with a DS = 70.6%, shows higher *IEC* and $\sigma_{p}$ values than the MUFABA34 membrane that shows a DS = 72.2%. The polyamide MUFASA34 is the one that contains the highest number of sulfonic acid groups of both series of new polyamides. Due to the latter reason, it also shows the highest *IEC* and *W_u_* values, which in turn is reflected in the highest proton conductivity of all the materials synthesized in this study.

Various efforts have been made to develop sulfonated polymeric materials with a sustainable approach around the world [36,37,38,39,40]. Table 4 shows some of these materials, along with their ionic properties, and compares them with the values obtained for the muconic acid-based polymers developed in this research. The results obtained for the sulfonated polymers MUFABA34 and MUFASA34 are comparable with other fully or partially renewable polymers bearing sulfonic acid groups. The latter indicates that the sustainable methodology addressed in this study is an effective tool to offer special environmentally friendly polymers.

## 4. Conclusions

The muconic acid monomer reacted successfully in polycondensation reactions with two aromatic-sulfonated diamines and a fluorinated diamine as comonomers for the synthesis of two series of partially renewable aromatic–aliphatic polyamides with an increasing degree of sulfonation (*DS*). *FTIR* and *NMR* spectroscopy methods were employed to confirm the polymer chemical structures and also revealed that the *DS* was effectively tailored by adjusting the feed molar ratio of the diamines. It was also found that the *W_u_*, *IEC*, and *σ_p_* values increased together with the DS. The highest value of *σ_p_* determined by electrochemical impedance spectroscopy (*EIS*) was found to be 9.895 mS·cm^−1^ at 30 °C corresponding to the MUFASA34 polyamide. The ionic properties reported in this study for the partially renewable muconic acid-based ionomers were close to other fully or partially renewable sulfonated polymers. It is important to note that the incorporation of renewable monomers is a powerful tool to synthesize sustainable and eco-friendly specialty polymers that can be an alternative for PEM applications.

## Figures and Tables

**Table 1:** Molar relations of monomers in the feed for the synthesis of the muconic acid-based polyamide series.

Polymer	MUA ^a^ (mmol)	HFDA ^b^ (mmol)	DABS ^c^ (mmol)	DASDA ^d^ (mmol)
MUFA	0.64	0.64	0.00	0.00
MUFABA14	0.64	0.48	0.16	0.00
MUFABA24	0.64	0.32	0.32	0.00
MUFABA34	0.64	0.16	0.48	0.00
MUFASA14	0.64	0.48	0.00	0.16
MUFASA24	0.64	0.32	0.00	0.32
MUFASA34	0.64	0.16	0.00	0.48

^a^ Muconic acid, ^b^ 4,4′-(hexafluoroisopropylidene)bis(*p*-phenyleneoxy)dianiline, ^c^ 2,5-diaminobencensulfonic acid, ^d^ 4,4′-Diamino-2,2′-stilbenedisulfonic acid.

**Table 2:** Physical properties of the muconic acid-based polyamide series.

Polymer	*T_g_* (°C) ^a^	*T_d_* (°C) ^b^	*d_i_* (Å) ^c^	*ρ* (g·cm^−3^) ^d^	*η_inh_* (dL·g^−1^) ^e^
MUFA	241	322	2.55	1.38	0.352
MUFABA14	244	318	2.05	1.38	0.343
MUFABA24	240	303	2.27	1.39	0.304
MUFABA34	241	273	1.85	1.43	0.348
MUFASA14	217	287	1.95	1.37	0.266
MUFASA24	229	272	1.97	1.38	0.213
MUFASA34	232	276	2.05	1.45	0.201

^a^ Glass transition temperature determined by *DSC*. ^b^ Decomposition temperature determined by *TGA*. ^c^ Average separation distance between polymer chains determined by *XRD*. ^d^ Density determined by the flotation method. ^e^ Inherent viscosity at a polymer concentration of 0.2 g·dL^−1^.

**Table 3:** Ionic properties of novel muconic acid-based polyamides.

Polymer	*DS* ^a^ (%)	*IEC* (mmol·g^−1^)		W_u_ (%)	$\sigma_{p}$ (mS·cm^−1^) ^c^
		Theoretical	Experimental ^b^		
MUFA	-	-	-	4.51	-
MUFABA14	24.3	0.43	0.37	7.87	0.027
MUFABA24	48.7	1.01	0.96	29.17	0.426
MUFABA34	72.2	1.82	1.80	33.49	1.608
MUFASA14	22.8	0.80	0.72	12.45	0.367
MUFASA24	46.1	1.70	1.80	28.78	0.849
MUFASA34	70.6	2.73	2.81	36.93	9.895

^a^ Determinated by ^1^H-*NMR*. ^b^ Determined by titration. ^c^ Determinated by *EIS*.

**Table 4:** Ionic properties’ comparison of the muconic acid-based ionomers reported in this study and some renewable polymers reported in the literature.

Polymer	*IEC*_Exp_ (meq·g^−1^) ^a^	*W_u_* (%) ^b^	*σ_p_* (mS·cm) ^c^	Reference Author
(SPEAE) ^d^	0.356	51.2	0.018	[36]
S5 ^e^	-	-	0.005	[37]
DASA ^f^	2.2	44.1	0.5	[38]
CH ^g^	0.37	249	2.44	[39]
DAFASA2/4 ^f^	0.8	17.1	1.5	[38]
LS 1.6 ^h^	1.6	25.9	8.12	[40]
MUFABA34	1.80	33.49	1.608	This study
MUFASA34	2.81	36.93	9.895	This study

^a^ Ion-exchange capacity determined by titration. ^b^ Water uptake determined by gravimetric measurements. ^c^ Proton conductivity determined by *EIS*. ^d^ Sulfonated poly(eugenolco-allyleugenol). ^e^ Chitosan-based membranes containing acetic acid (HAC) and plasticized with glycerol. ^f^ Oleic acid-based polyamide. ^g^ Pristine chitosan. ^h^ Ionomer from kraft lignin.

## Data Availability

The data presented in this study are available from the corresponding authors upon reasonable request.
